# Coastal erosion management in Accra: Combining local knowledge and empirical research

**DOI:** 10.4102/jamba.v8i1.274

**Published:** 2016-11-18

**Authors:** Kwasi Appeaning Addo, Irene Appeaning Addo

**Affiliations:** 1 Department of Marine and Fisheries Sciences, University Of Ghana, Ghana; 2 Institute for African Studies, University of Ghana, Ghana

## Abstract

Coastal erosion along the Accra coast has become a chronic phenomenon that threatens both life and property. The issue has assumed a centre stage of national debate in recent times because of its impact on the coastal communities. Lack of reliable geospatial data hinders effective scientific investigations into the changing trends in the shoreline position. However, knowledge about coastal erosion, by the local people, and how far the shoreline has migrated inland over time is high in the coastal communities in Accra. This opens a new chapter in coastal erosion research to include local knowledge of the local settlers in developing sustainable coastal management. This article adopted a scientific approach to estimate rate of erosion and tested the results against perceived erosion trend by the local settlers. The study used a 1974 digital topographic map and 1996 aerial photographs. The end point rate statistical method in DSAS was used to compute the rates of change. The short-term rate of change for the 22-year period under study was estimated as -0.91 m/annum ± 0.49 m/annum. It was revealed that about 79% of the shoreline is eroding, while the remaining 21% is either stabilised or accreting. It emerged, from semi-structured interviews with inhabitants in the Accra coastal communities, that an average of about 30 m of coastal lands are perceived to have been lost to erosion for a period of about 20 years. This translates to a historic rate of change of about 1.5 m/year, which corroborates the results of the scientific study. Again this study has established that the local knowledge of the inhabitants, about coastal erosion, can serve as reliable information under scarcity of scientific data for coastal erosion analyses in developing countries.

## Introduction

Coastal erosion management strategies have evolved over the years in tandem with technological advancement. Such management approaches have depended mainly on monitoring empirical measurement of past and present shoreline changing positions, as well as projecting the future trends under changing climatic conditions. However, using only empirical measurement as base information in developing coastal management strategies may not be inadequate. This is because such activity excludes important information that may not be captured but could be significant in managing coastal erosion. There is, therefore, the need for the integration of local or traditional knowledge in coastal erosion management. This, according to Alexander *et al*. (2011), will give a human face to such a management approach, as it will make the communities feel part of such systems. Such integration offers enough data base in coastal regions with limited available geospatial data. This article investigates the possibility of using both scientific and local knowledge to sustainably manage coastal erosion in Accra.

Coastal zones support a wide range of unique habitats and species, as well as economies of coastal nations globally in the areas of agricultural productivity, fishing industries, tourism development and commerce (Al-Tahir & Ali 2004; Hall 2001). The shoreline status is therefore critical to the coastal population, wildlife and coastal assets. Coastal regions are also locations of major industries and are inhabited by approximately 50% of the global total population (Woodroffe 2003), which is predicted to increase by 32% in 2025 (Duedall & Maul 2005). Coastal erosion problems continue to be a considerable challenge to coastal communities and governments as coastal resources and infrastructures are under severe threat. Such threats have increased over the past two decades (Hays, Richardson & Robinson 2005), where it is estimated that about 70% of the world’s sandy beaches are eroding (Bird 2005).

Coastal erosion is also a major threat in West Africa. This threat is evident in Ghana coastline. According to EPA (2003), coastal erosion in Accra has become a key environmental issue in Ghana. The Accra coast has monumental infrastructure such as the Christianborg Castle, Kwame Nkrumah Mausoleum and the Independence Square. These monumental infrastructures epitomise the history of Ghana by providing a link between the nation’s past, present and future. In addition, the Accra coast is experiencing increased infrastructure development that is expected to affect the dynamics of the coastal system. It is estimated that about 80% of the Accra shoreline is eroding (Appeaning Addo, Walkden & Mills 2008). This has affected sources of livelihood and displaced settlements in the coastal communities (AMA 2006; Appeaning Addo 2009a; Campbell 2006; Sagoe-Addy & Appeaning Addo 2013). The situation calls for a holistic management approach. Unfortunately, mainly scientific data have been used to design coastal structures without addressing the coastal erosion impact on livelihoods. The question that has been a subject of intense debate in scientific circles is the role of local or traditional knowledge in bridging the planning gap (Dolan & Walker 2003; Oteng-Ababio, Owusu & Appeaning Addo 2011; Twinomugisha 2005). However, there is very little documentation on the use of local and traditional knowledge in coastal erosion management in data-starved developing countries. Studies by Adger, Arnell and Tompkins (2005) concluded that community perceptions of coastal erosion have only rarely been studied and even more rarely used as a contribution to management. Another gap is the combination of local knowledge with scientific research in coastal erosion management. This, according to De Freitas and Tagliani (2009), has resulted in ineffective development and implementation of management policies to address coastal problems because of a failure to use all available sources of information and knowledge. The integration between traditional and scientific information can fill gaps in technically generated data and hence produce scientifically valid and locally relevant information (De Freitas & Tagliani 2009). This article will highlight the role of the local community in understanding issues of coastal erosion in Accra.

## Coastal erosion management

### Traditional or local knowledge use in coastal erosion management

Traditional or local indigenous knowledge is described as the cumulative body of wisdom, knowledge and practices of indigenous people gained over time through experience, evolving by adaptive processes and orally passed on from generation to generation (Ajibade 2003; Salick & Byg 2007). Such knowledge enables scientific views of changes to be framed in a local context, provides a platform to ‘ground truth’ scientific research, allows for better local examination, expression and interpretation of global changes (Riedlinger & Berkes 2001; Usher 2000) and provides improved foundations for decision-making and adaptive capacity building (Dolan & Walker 2003). Local indigenous knowledge has been applied in fisheries management, pollution management, forestry management, mangrove management and disaster risk reduction (Hiwasaki *et al*. 2014). Some traditional knowledge and management systems have been used to interpret and respond to feedback, which has addressed uncertainty and unpredictability in ecosystems’ research (Berkes, Colding & Folke 2000). The contribution of local and traditional knowledge to understanding climate change in remote regions is well documented (Bielawski 1995; Cohen 1997; Fast & Berkes 1998). Hiwasaki *et al*. (2014) have suggested:

Local and indigenous knowledge (LINK) is often best sustained in traditional communities which are family oriented. This knowledge is a way of projecting the identity of a community, and the wealth of LINK reflects their culture and identity, and can speak a lot about a society’s social system such as obligatory reciprocity and relationships with the divine. Having an established identity and unity is a community resource that can also be harnessed as a resource for disaster risk reduction (DRR) and climate change adaptation (CCA). (p. 31)

Local indigenous knowledge of coastal erosion in coastal communities varies from one geographical location to another. These variations are driven mostly by the level of understanding of the shoreline changing dynamics over a period. Causes of shoreline change have been grouped into three areas (Shaghude *et al*. 2015). According to the authors, the first is the physical forcing which includes rainfall, wind, local waves, swell waves, tidal currents, sea level rise and storm surges. The second is the natural resources available including sand supply, the geology and geomorphology of the coast. The third is from anthropogenic activities of man including sand mining and introduction of coastal protection elements. Shoreline change affects the social, structural, economic infrastructure and environmental condition of a coastal community (Shaghude *et al*. 2015). Using scientific method, the physical forcing and the natural resources that influence shoreline change in a coastal community are determined. Similarly, the scientific method may be able to estimate the effect of shoreline change on the structural buildings, infrastructure and the environmental situation including mangroves and beaches. However, using local knowledge, information can be gathered on the social and human dimension of the causes and risks associated with shoreline change.

The local knowledge, influenced by the coastal dwellers familiarity with the coastal environment over a relatively long time and displacement of households in the communities, is an important management resource that cannot be overlooked. An example of research combining both the indigenous knowledge and the scientific research is Alexander *et al*. (2011). The authors linked indigenous knowledge to scientific knowledge by collecting 57 indigenous narratives describing climate change and its impact and linking it to scientific research on a large scale in the northern Hemisphere. The study revealed that the two knowledge systems complement each other and that indigenous knowledge can provide complementary information about climate change in determining patterns for regions in which there are limited instrumental records. Again, it emerged from the study that indigenous knowledge promotes an expanded and multidimensional picture of the impacts of climate change by placing the changes in the context of a human landscape (Alexander *et al*. 2011). Studies by Pascal *et al*. (2011) used an integrated approach comprising local knowledge, LIDAR surveys and a DGPS system to map the levels reached because of flooding during a storm event in Canada in 2005. The study, with the help of the local knowledge, identified that such levels vary greatly in spatial terms and that the difference between the levels actually reached and the water level measured by tide gauge can be as much as 2 m. A scenario-building exercise in Costa Rica, undertaken as part of the Millennium Ecosystem Assessment (Bennett & Zurek 2006; Parry 2007), identified the potential for joint scenario-building incorporating different forms of knowledge systems. Incorporating indigenous knowledge into scientific knowledge can lead to the development of effective adaptation strategies that are cost-effective, participatory and sustainable (Mercer *et al*. 2007; Robinson & Herbert 2001).

### Coastal erosion management in Ghana

According to Amlalo (2005), there is no specific coastal erosion management policy in Ghana, although there are several environment-related policies that address the protection, management and development of the marine and coastal environment. These policies are supposed to promote integrated coastal zone management and sustainable development. Currently, the management of the coast is partly subsumed under the National Environmental Policy and it is being coordinated by the Ministry of Environment, Science and Technology (MEST) and the Environmental Protection Agency. Under this policy, the environmental challenge identified is marine and coastal degradation. The characteristics associated with this challenge include the following:

- Marine and coastal areas are under pressure due to: intensive agricultural production, industrial development, salt production, mining and quarrying and urban development.
- Sources of pollution are municipal and industrial effluents, agricultural runoffs.
- Sea erosion at Keta and Ada (and other places).

The proposed management options include:

- Direct investment in control structures, for example, Keta Sea Defence Project.
- Gabions and boulder revetments to arrest erosion.
- Mangrove replanting and planting of other vegetative cover, example at Winneba.
- Regulatory incentives or fines for illegal mining.
- Policy reforms in land use planning and coastal zone management.
- Investments in waste treatment and small-scale waste collection.

As the developmental priority of Government is centred on uneconomic development concept for fishery and industry, beach revetment and groynes are usually the preferred option. According to Addo *et al*. (2008), coastal erosion management in Ghana is reactive, site-specific and usually involves the use of hard engineering approaches. These approaches, which tend to build against nature, stabilise the shoreline at the protected section but increase erosion elsewhere (knock-on effect). These approaches are unsustainable and environmentally unfriendly. According to Boateng (2009), coastal erosion management has been done on an ad hoc basis because of lack of a comprehensive management scheme. Communities usually complain about destruction of properties and the government goes in to offer these mitigation measures. Lack of a comprehensive management policy has prevented developing a sustainable strategy in managing coastal erosion holistically. Major sea defence project have been carried out at sites considered to be highly vulnerable including the Keta Sea Defence Project, Ada Sea Defence Project, Sakumono Sea Defence Project, New Takoradi Sea Defence Project, among others. The Keta Sea Defence Project is reported by Angnuureng, Appeaning Addo and Wiafe (2013) to have increased coastal erosion on the downdrift coast.

## Study area

**FIGURE 1:**
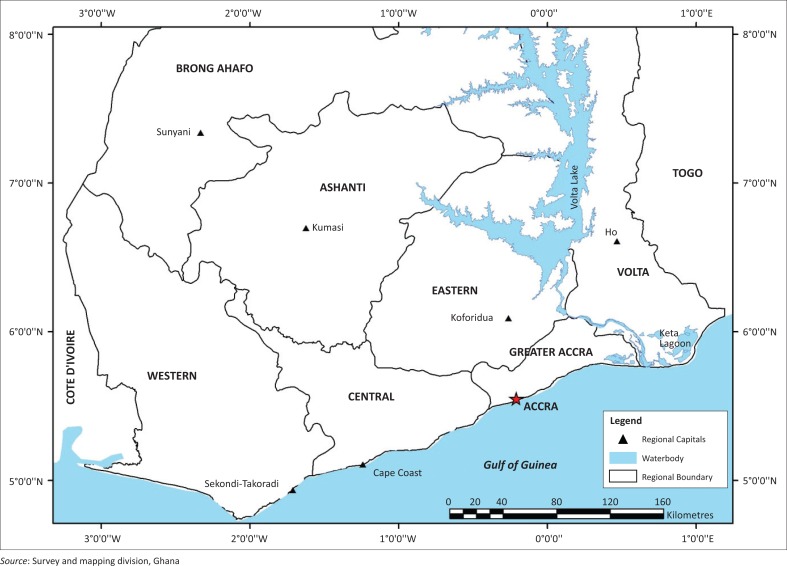
Location of the study area along the Ghana coast.

Accralies, along the Gulf of Guinea, at latitude 5.626°N and longitude 0.1014°W (Figure 1) influences the climatic conditions that prevail along the coast. It is the political and economic capital of Ghana. The shoreline is about 40 km long. The coastal zone is generally a low-lying area with successions of ridges, slopes and occasional rocky headlands. The elevation ranges between 1.5 m and 12.0 m (Appeaning Addo 2009b). Along the shoreline, sandy platforms are associated with lagoonal inlets, and these platforms consist of unconsolidated sands with occasional interlayers of silt and clay. The coastal area experiences bimodal rainy seasons with the major season between April and July, and the minor one between September and November. Sediment transport to the littoral zone is high during the rainy season as the rivers discharge their sediment from the upland catchment areas into the sea. Inversely, sediment transport reduces during the dry season when temperatures are over 30 °C resulting in the drying up of most of the rivers.

Waves approach the open coast from the south–southwest direction (Angnuureng *et al*. 2013). The significant wave height is about 1.4 m and the period is from 10 to 15 s. Currents that transport sediment include the long shore current that varies between 0.5 m/s – 1.5 m/s, the Guinea current that can measure up to 0.5 m/s during the rainy season but is weak most of the year and the tidal current that plays no significant role in the coastal morphology (Wellens-Mensah *et al*. 2002).

Erosion has affected the social and economic life of the local population, threatened cultural heritage and hindered coastal tourism development. In the western part of the Accra coast, 17 coastal inhabitants have lost their buildings to coastal erosion over 26 years (Campbell 2006). Coastal retreat has also eroded natural fish landing sites, collapsed the copra business (manufacturing of coconut oil) and degraded the coastal environment. Erosion has therefore affected both the local and national economy. Accra’s shoreline recession has been the subject of previous studies which identified both natural and human activities as the cause of erosion (Appeaning Addo *et al*. 2008; Sagoe-Addy & Appeaning Addo 2013). Poor decision-making on infrastructure location, inappropriate mitigation measures, resource extraction and variable climatic conditions are some of the major causes of shoreline erosion. Non-involvement of local coastal residents in coastal management activities and the disjointed measures in solving perceived erosion problems have resulted in inconsistent approaches in coastal erosion management in Accra. The lack of appropriate coastal policies as identified by Amlalo (2005) and Boateng (2009) has prevented vertical and horizontal integration in decision-making concerning the coastal zone.

## Methodology

The research was in two parts. The first part used empirical data including aerial photographs and topographic maps to identify the occurrence of erosion, determine the trend and extent of shoreline inland migration, and estimate the historic erosion rates. The second part of the study used semi-structured questionnaires to elicit for inhabitants local knowledge and perception on coastal erosion in the communities.

### Geospatial determination of coastal erosion

**FIGURE 2:**
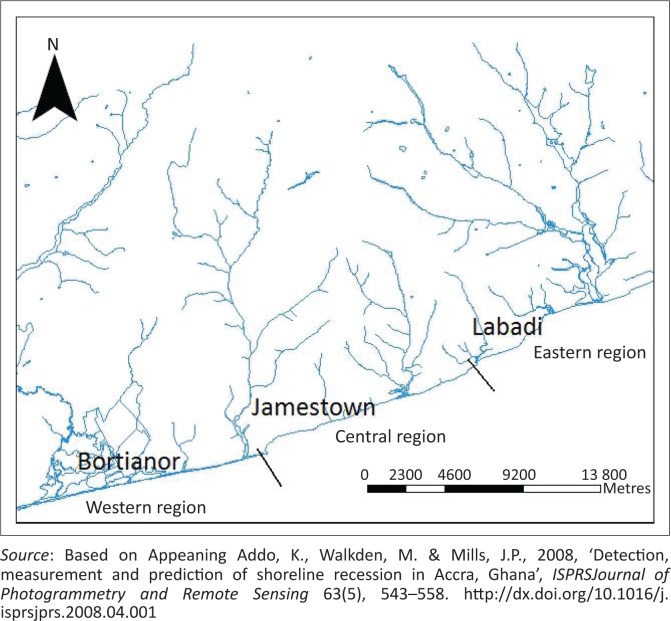
Division of Accra coast into three geomorphic regions.

The study area was zoned into three regions, namely western, central and eastern based on the geomorphology (Figure 2). Previous studies identified that the western and eastern regions are eroding more in the long term relative to the central region (Anokwa, Martin & Muff 2005; Appeaning Addo *et al*. 2008; Campbell 2006). Thus the anticipated impact of coastal erosion is expected to be more in the western and eastern sections.

**FIGURE 3:**
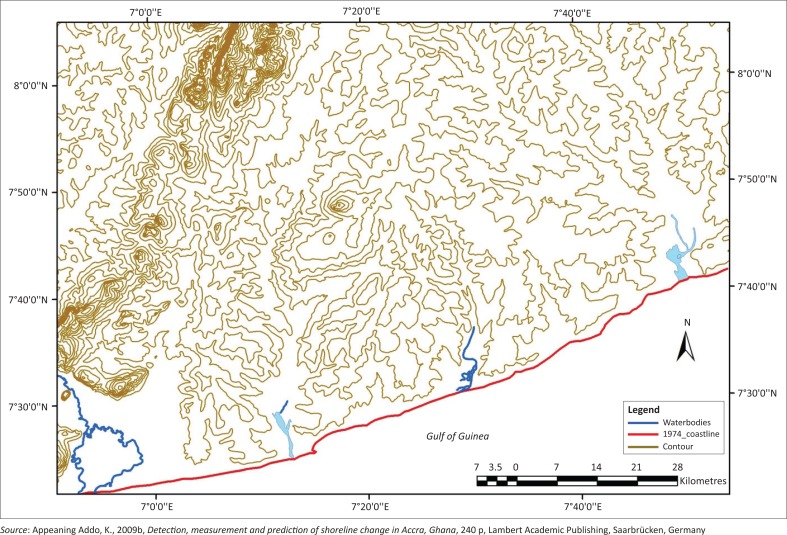
1974 Topographic map of Accra showing the shoreline position.

Empirical data for the study included a 1974 topographic map and 1996 aerial photographs obtained from the Survey and Mapping division of the Lands Commission of Ghana. The reliability of the 1974 digital topographic map has been determined by various studies (Appeaning Addo *et al*. 2008; Wiafe *et al*. 2013). This increased confidence in using the data for this study. A further shoreline positional accuracy check was done and the sources of uncertainty identified were subsequently quantified. Figure 3 is a section of the 1974 topographic map showing the shoreline position in Accra which was extracted for the rates of change analysis. The 1996 aerial photographs were georeferenced using the 1974 topographic map as the base map. The data were exported to ArcGIS using the Ghana meter projection. This ensured compatibility and facilitated comparing the shorelines to detect change. The shoreline positions on the topographic map and the aerial photographs were carefully digitised so as to reduce introducing error that could affect the reliability of the results and saved separately as shape files.

The 1974 topographic map was generated from aerial photographs using analogue photogrammetric methods. The plausible sources of errors include digitising error and photogrammetric mapping error. The source of error associated with the 1996 data is mainly digitising error. Error as a result of cartographic pen was neglected in both cases since the shoreline data were in digital format. The HWL proxy was used to represent the shoreline positions on all the data (Appeaning Addo *et al*. 2008). Various studies (Crowell *et al*. 1991; Moore 2000; Morton, Miller & Moore 2004; Thieler & Danforth 1994) provide estimates of typical measurement errors associated with mapping methods and shoreline digitising, and these were used to inform this study. The total shoreline position error was estimated using the equation:

$$E_{n}\;=\;\sqrt{\left(E_{s}^{2}\;+\;E_{d}^{2}\;+\;E_{p}^{2}\right),}$$ [Eqn 1]

where *n* = shoreline number, *E*_s_ is the error occurring from scale difference, *E*_p_ is the photogrammetric error and *E*_d_ is the digitising error. This approach carries the assumption that component errors are normally distributed (Dar & Dar 2009).

A separate total error (*E_n_*) was calculated for each shoreline. The positional error for each period was then incorporated into an error for each transect. These values were annualised (*Ea*) to provide error estimation for the shoreline change rate at any given transect and expressed as:

$$E_{a}\;=\;\frac{\sqrt{\left(E_{1}^{2}\;+\;E_{2}^{2}\right)}}{T},$$ [Eqn 2]

where *E*_1_ *and E*_2_ are the total shoreline position error for the two different years and *T* is the 22-year period of analysis. The maximum annualised uncertainty using best estimate for this study is ±0.49 m/year. The shoreline position on the 1974 topographic map and the 1996 aerial photographs were carefully digitised to reduce the level of subjectivity in delineating the shoreline in ArcGIS. The 1974 map was transformed from imperial to metric units to ensure compatibility with the 1996 data which were already in metric units. The shoreline positions were compiled and managed in ArcGIS. A geodatabase was created for the extracted shoreline positions and each shoreline has attributes such as date, length, ID, shape and uncertainty. The dates of the data were manually entered in the date column while the length, ID and shape were automatically generated. The quantified uncertainties were also entered as integers for the uncertainty column. The two shoreline positions were then appended to one shapefile for rate calculation. The Digital Shoreline Analysis System (DSAS) developed by the US Geological Survey (USGS) (Himmelstoss 2009) was used for rate estimation. The software is an extension for ArcGIS and computes rate of change at user specified interval along the shoreline using different methods. It uses the measurement baseline method to calculate rate of change statistics for a time series of shorelines. The baseline is constructed to serve as the starting point for all transects cast by the DSAS application. For this analysis, the baseline was constructed by manually digitising about 300 onshore away from the closest shoreline by mimicking the general orientation of the outer shoreline.

Once all the inputs were ready in the database, transects were automatically constructed after specifying a 100-m transect interval. A transect interval of 100 m was adopted for this study since, according to Doukakis (2004), transect spacing below 100 m does not result in improved estimates of shoreline change rate. In all, a total of 380 transects were cast along the entire stretch of about 40 km shoreline from west to east. Transects were cast at right angles from the baseline and the historic rates of shoreline change calculated at each transect using the end point rates (EPR). EPR is the most commonly used method to compute shoreline rate of change (Crowell *et al*. 2005; Genz *et al*. 2007). The method is simple and requires only two shoreline positions to obtain a rate of change. It calculates the rate of change by dividing the distance of shoreline movement by the time elapsed between the earliest and the latest measurement, which can be the oldest and the most recent shoreline positions.

### Semi-structured interviews

A semi-structured interview was conducted with about 50 respondents. The respondents were randomly sampled from the coastal communities in the study area. These communities are Bortianor, James Town and La. Thirty of the respondents were male while 20 were female. The respondents included fishers, petty traders, public servants, opinion leaders, the hospitality industries and district assembly members. Permission was sought from the local assembly man and the traditional leaders in the communities before interviews were conducted. This was due to their high level of indulgence in sand and gravel minning activities banned by the government and the fear of being reported to the police. Secondly, these leaders helped identify key respondents for the interviews. The respondents were all above 20 years. The oldest age was 80 years, an old man who has lived in the community his entire life. The semi-structured interview comprised of 20 questions. The first set of questions collected information on the demographics of the respondents. The second set of questions examined respondents’ perception of coastal erosion and the third set of questions looked at coastal erosion impact on the social and economic livelihoods of the respondents. The last set of questions looked at sand mining activities and mitigation of coastal erosion (Table 1).

**TABLE 1:** Semi-structured questionnaire.

Period for change detection	Number of respondents	%
Over 20 years	5	10
Between 10 and 15 years	20	40
About 10 years	15	30
Cannot tell	10	20

After the semi-structured interview, 10 key persons were selected for an in-depth discussion. These set of respondents were used to further clarify information gathered on detection of coastal erosion and its impact on their communities in the past 20 years. Hence, households who were above 40 years and had lived in the community for more than 20 years were interviewed. Preliminary studies identified erosion to be severe in the western (Bortianor) and eastern (La) regions relative to the central region (James Town). Hence, only 10 persons were interviewed in James Town, while 20 persons were interviewed in Bortianor and another 20 persons interviewed in La.

## Results

### Shoreline recession in the study communities

**FIGURE 4:**
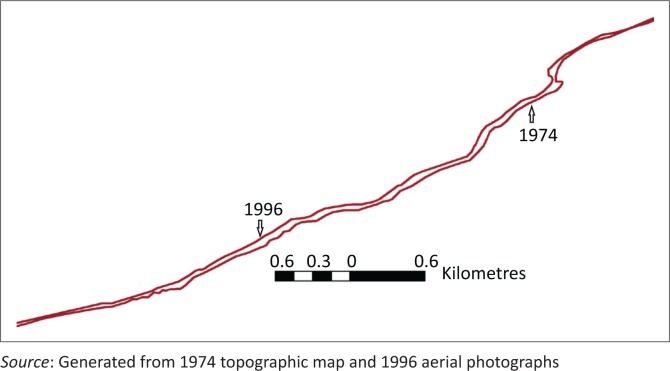
Merged 1974 and 1996 shoreline positions.

Merging the two shoreline positions in ArcGIS enabled the eroding areas to be identified (Figure 4). It showed that the shoreline has migrated inland from 1974 to 1996 at varying intensity alongshore. The orthogonal transects to the baseline enabled how far inland the shoreline position has moved over the 22-year period.

Although the period under study, 22 years, is relatively short and can therefore be considered as short term for this study, the change computed, however, gives indication of the likely trend in the long term if prevailing climatic conditions experience minimal variation. Short-term rates of change were computed for each of the 387 transects using the EPR method. The estimated shoreline rate of change as well as the percentage of coastal land eroding/accreting during the period under study is tabulated in Table 2. The identified uncertainty was quantified as ± 0.49 m/year.

**TABLE 2:** Estimated short-term rates of change.

Period	Mean erosion rate (m/yr)	%	

		Erosion	Accretion
1974–1996	-0.91 ± 0.49	79	21

*Source*: Computed from 1974 topographic map and 1996 aerial photographs

Rates of change were also estimated separately for each of the three geomorphic regions to facilitate comparing erosion trend and intensity in the three regions. The results are tabulated in Table 3.

**TABLE 3:** Estimated short-term rates of change in the three geomorphic regions.

Period	Mean erosion rate (m/yr)		

	Western region	Central region	Eastern region
1974–1996	-0.35 ± 0.49	-0.82 ± 0.49	-1.74 ± 0.49

*Source*: Computed from 1974 topographic map and 1996 aerial photographs

**FIGURE 5:**
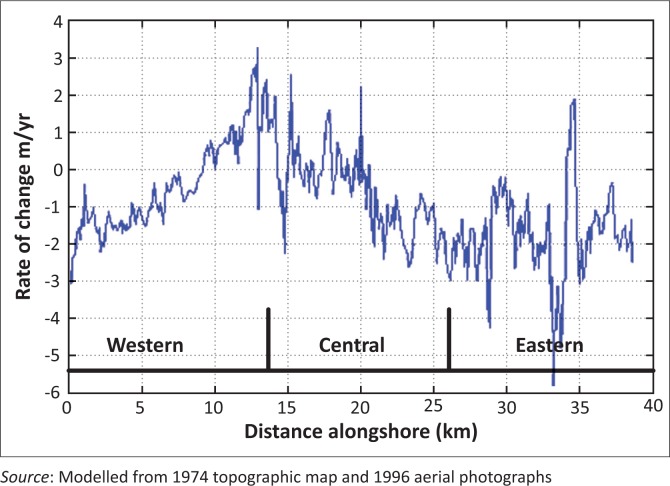
Shoreline recession trend along the coast.

The computed rates along transects were used to generate the shoreline evolution trend along the Accra coast (Figure 5).

### Local knowledge and perception

#### Demography

The respondents were made up of about 20 females and 30 males. From the results, 20 persons had lived in the community for more than 20 years, 15 persons had lived in the community between 30 and 40 years, 10 persons had lived in the community between 20 and 30 years and only 5 persons had lived in the community for less than 20 years. This increased confidence in the reliability of the response.

#### Coastal erosion in the communities

It emerged from the results that almost all the respondents had noticed significant changes in the shoreline position. From the results, 5 persons first observed coastal erosion 20 years ago, 20 persons observed erosion between 10 and 15 years ago, 15 persons observed the coast eroding 10 years ago and 10 persons could not tell whether there was any occurrence of coastal erosion.

With regard to the use of the beach, the respondents mentioned that they were using the coast for different activities. While 20 persons claimed they traded on the coast, 25 persons said they were fishermen and claimed that the coast was their landing site. The remaining 5 persons mentioned that they used the beaches for recreation. Some of the trading activities included selling fresh fish, smoking and drying fish, selling premix fuel for and food vending. One respondent intimated that business activity on the shore had decreased considerably in their communities but increased in the adjoining communities because the fishermen were able to land their fish catch on those beaches. Others responded that since the shore started eroding, some of the fishermen have abandoned the communities and are now moving to neighbouring communities. The shores of those communities are also becoming overcrowded. For example, the landing beach at the Fire Service Training School in James Town eroded so much that it hampered the activities of the fishermen. According to the people, the fishermen now land their catch in La. The few fishermen who continue to land their fish catch in the communities complained of lack of adequate space and lack of shade from coconut trees. While half of the respondents attributed the loss of coconut trees to coastal erosion, the remaining suggested that the coconut trees became extinguished due to the coconut disease that afflicted the communities some years ago.

With regards to eroding buildings at the coast, only 5 respondents could confirm that the coastal erosion had affected buildings in the communities. The respondents explained that some of the affected families had migrated from the communities while the majority have moved inland to stay with extended family members. The communities are built up and there are no reserved lands for new development.

#### Sand and gravel mining

Thirty of the respondents confirmed that sand and gravel mining is still ongoing even though the government had banned such activities. However, 15 persons said the activity had stopped and 5 persons mentioned that they were not aware of any sand and gravel mining activity. From a cursory look, it was observed that sand mining was still going on.

#### Mitigation measures

According to 10 of the respondents, there has been some attempt to stop coastal erosion in their communities. However, the remaining 40 respondents claimed that no attempt had been made to stop coastal erosion in their communities. Observations made, indicate that coastal reclamation had been made in only James Town. This explains why the 10 respondents from James Town confirmed that there were mitigation efforts in their community. While seven out of ten respondents claimed that the coastal reclamation had been successful, three were of the view that the reclamation exercise was not effective because they still see the coast eroding.

## Discussion

It emerged from the survey that erosion has affected households and the family systems considerably. Families displaced by coastal erosion have either left the communities or are now staying with friends and relations. Almost all the respondents identified the fishing industry to have been seriously affected by coastal erosion. Declining fish catch as a result of climate change and eroding natural fish landing sites were the major issues identified to be confronting fishing activities in the communities confirming what was scientifically observed by Nunoo and Asiedu (2013). This has resulted in migration of the fishers to nearby communities, to neighbouring countries and introduced economic stress on households. Some have developed alternative sources of livelihood such as beach sand mining for the construction industry. Although this activity is banned in Ghana (Jonah 2014), about 60% of the respondents identified the activity to be on going.

Migration of fishers to other fishing communities have also resulted in overcrowding, which has increased stress on social amenities and reduced patronage of food vending along the beaches. It emerged from the structured interviews that fishers are increasingly experiencing difficulties in landing their catch due to lack of space. Some have to travel long distances from their communities to land their catch. The coconut business used to be one of the major sources of employment in the study area according to the survey. However, 80% of the respondents indicated that the business has collapsed and resulted in increased unemployment and economic hardship. The destruction of the coconut vegetation, which used to serve as shelters for the fishers to mend their nets, has exposed them to the direct rays of the sun during net mending with its related health implications. Residents have now turned to sand mining as an alternative livelihood. One resident said during the interview that ‘once the sand is mined, the sea has abundant store of sand and will refill the gap with more sand’. Another said ‘if the government provides us with jobs we will stop beach sand mining’. Beach sand mining has become attractive because of the decline in the two major sources of employment, fishing and the coconut business. According to Campbell (2006), the collapse of the coconut groves along the coast is due to disease, over cutting, settlement expansion and coastal erosion.

The local knowledge system, which the indigenous people gain over time through experience, is influenced significantly by the length of stay in a particular environment. The longer a person stays in a particular environment, the more familiar he or she identifies changes within the environmental settings. It emerged from the questionnaires sent out that about 70% of the respondents have lived in the study area for over 30 years. This is considered long enough to observe changes in the shoreline system since they are always in physical contact with the coastal environment. Therefore the results obtained from the survey can be considered reliable. This is because it is expected that the local people would have acquired a cumulative body of insight through experience in the changing trends in the dynamic shoreline system.

The survey results indicate that almost all the respondents have noticed significant changes in the shoreline position. Settlers in the past sited their buildings away from the beach front (Hays, Richardson & Robinson 2005). Coastal erosion was not considered a problem in the past since it did not directly affect settlement. However, in recent times development is moving more towards the shoreline position due to increased coastal population and government policy on coastal tourism (Appeaning Addo 2013). Previously very few houses were destroyed through coastal erosion collaborating scientific studies by Campbell (2006), who identified that about 17 coastal buildings in the western part of Accra have been destroyed by erosion within a period of 26 years, which is on the low side. It is therefore not surprising that a majority of the respondents claimed that they had not observed any building destroyed through coastal erosion. Although there has been an attempt to manage coastal erosion, such measures have not always been successful in some locations. The respondents observed that the interventions might be the cause of increased erosion along other portions of the coast. Angnuureng *et al*. (2013), observed that the introduction of coastal defence in Keta is causing the downdrift communities to experience increased coastal erosion in the Volta region.

Local dwellers’ perception about how far inland the shoreline has moved is influenced by relating such movement to either an event or a land mark. Although such observations may be skewed and not accurate, they can facilitate deducing semi-quantitatively the evolution trends in the shoreline position over a period. An 80 year old recounted his past when he used to play football on the beach (about 50 meters into the sea from the present shoreline position) during his youthful age but claims that now everything has been taken over by the sea. Another resident in La said ‘my ancestral home is over there (approximately 35 m) buried deep in the ocean’. A 45-year-old lady said during the interview that ‘my father told me they used to walk about 8 poles (about 40 m) to get to the shoreline’. Deductions from the results of the interview of some of the inhabitants suggest that the shoreline has moved inland by about 30 m since the last 20 years. This translates to approximately 1.5 m/yr, which is quite high. However, the computed short-term rate for the period between 1974 and 1996 is about -0.91 m/yr ± 0.49 m/yr (refer to Table 3). The upper range of this estimate, -1.40 m/yr is close to the perceived rate from the local knowledge. This suggests that local knowledge about coastal erosion trends in the Accra coastal communities is credible. The information can be used for developing management strategies in locations where there is a lack of shoreline geospatial data. Although the reliability of the estimated rates of change using local knowledge systems cannot be checked statistically, it can however be used to validate modelled rates of shoreline change results for data-starved regions.

## Conclusion

Coastal erosion along the Accra coast was obvious to the respondents from the communities, and this was confirmed using the scientific method. The local knowledge was able to provide cursory outlook of the coastal erosion situation in the communities. However, the community could not determine exactly the land loss within a spate of 20 years. Land loss was calculated through empirical research. This is necessary to inform planning purposes and introduce setback lines at the coast. There is therefore the need to calculate the rate of erosion through scientific means. However, the impact of coastal erosion on the communities can be ascertained through the semi-structured interviews. Respondents were able to assess the impact of coastal erosion on their livelihoods and what the implications are for families. Again they were able to confirm that migration was on going in communities with eroded coasts.

According to Niang *et al*. (2012), local communities living on the coast need to have full control of the coastal situation in order to select and apply appropriate adaptation measures. Working with the local people can provide warnings about existing problems before they become serious (Niang *et al*. 2012). This can be achieved if local knowledge is combined with scientific knowledge. Local knowledge can be a very useful tool in developing sustainable coastal erosion management strategies for data-starved coastal nations. In summary, the research identified four reasons why local knowledge should be combined with scientific data to achieve sustainable management of the Accra coast. They are:

- Inadequate data; local knowledge can provide cursory information on the coastal erosion situation.
- Calculate the land lost to erosion; the scientific data can provide the information statistically.
- Determine the rate of erosion; the scientific data can be computed to give this information.
- Determine the impact of coastal erosion on livelihoods; this information can be collected through interviews with the local community.

It is recommended that a coastal erosion management policy for Ghana should be developed. Again, coastal planning guidelines for the Accra coast should include the introduction of building setback distances, creating awareness and educating the coastal inhabitants on the causes and effect of shoreline change on the communities (UNESCO 2003). Combining local and scientific knowledge systems can help develop a system that can include the local people in co-managing coastal erosion. The local community can even be introduced to skills, training and tools for beach-change data collection, analysis, interpretation and application (UNESCO 2003). Such a system can result in sharing responsibilities in managing coastal erosion in Ghana. This will eventually result in a robust coastal erosion monitoring scheme for data-starved and less-resourced coastal nations.
